# Altered dynamic functional network connectivity in drug-naïve Parkinson’s disease patients with excessive daytime sleepiness

**DOI:** 10.3389/fnagi.2023.1282962

**Published:** 2023-12-06

**Authors:** Zhiyi Tan, Qiaoling Zeng, Xuehan Hu, Duoduo Di, Lele Chen, Zhijian Lin, Guanxun Cheng

**Affiliations:** ^1^Department of Medical Imaging, Peking University Shenzhen Hospital, Shenzhen, Guangdong, China; ^2^Department of Neurology, Peking University Shenzhen Hospital, Shenzhen, Guangdong, China

**Keywords:** Parkinson’s disease, excessive daytime sleepiness, resting-state functional magnetic resonance imaging, dynamic functional brain connectivity, neural network

## Abstract

**Background:**

Excessive daytime sleepiness (EDS) is a frequent nonmotor symptoms of Parkinson’s disease (PD), which seriously affects the quality of life of PD patients and exacerbates other nonmotor symptoms. Previous studies have used static analyses of these resting-state functional magnetic resonance imaging (rs-fMRI) data were measured under the assumption that the intrinsic fluctuations during MRI scans are stationary. However, dynamic functional network connectivity (dFNC) analysis captures time-varying connectivity over short time scales and may reveal complex functional tissues in the brain.

**Purpose:**

To identify dynamic functional connectivity characteristics in PD-EDS patients in order to explain the underlying neuropathological mechanisms.

**Methods:**

Based on rs-fMRI data from 16 PD patients with EDS and 41 PD patients without EDS, we applied the sliding window approach, k-means clustering and independent component analysis to estimate the inherent dynamic connectivity states associated with EDS in PD patients and investigated the differences between groups. Furthermore, to assess the correlations between the altered temporal properties and the Epworth sleepiness scale (ESS) scores.

**Results:**

We found four distinct functional connectivity states in PD patients. The patients in the PD-EDS group showed increased fractional time and mean dwell time in state IV, which was characterized by strong connectivity in the sensorimotor (SMN) and visual (VIS) networks, and reduced fractional time in state I, which was characterized by strong positive connectivity intranetwork of the default mode network (DMN) and VIS, while negative connectivity internetwork between the DMN and VIS. Moreover, the ESS scores were positively correlated with fraction time in state IV.

**Conclusion:**

Our results indicated that the strong connectivity within and between the SMN and VIS was characteristic of EDS in PD patients, which may be a potential marker of pathophysiological features related to EDS in PD patients.

## Introduction

Parkinson’s disease (PD) is the second most common neurodegenerative disease, with more than 8.5 million individuals suffering from PD worldwide (Grover et al., 2022). Excessive daytime sleepiness (EDS) is a frequent nonmotor symptoms in PD patients, affecting up to 20–60% of PD patients (Liu et al., 2022). EDS mainly manifests as the inability to stay awake and alert during typical waking periods, leading to unintended lapses into drowsiness or sleep states (Liu et al., 2022). EDS in PD patients not only has a negative impact on the patients’ quality of life (Bloem et al., 2021), including occupational, psychological, and social abilities, but may also worsen nonmotor symptoms such as mood, apathy, anxiety, and cognitive function (Chan et al., 2020). Therefore, there is a strong interest in identifying the neurobiological abnormalities underlying EDS in PD patients. A better understanding of the pathogenesis of EDS and the identification of its biomarkers are essential for PD patients.

Resting-state functional magnetic resonance imaging (rs-fMRI) is a novel noninvasive neuroimaging method with high temporal and spatial resolution that has been widely used to explore the pathogenesis of EDS in PD patients. The amplitude of low-frequency fluctuation (ALFF), fractional ALFF and regional homogeneity methods reflect the intensity and consistency of spontaneous activities among brain regions. Previous studies have displayed increases in spontaneous neural activity in the medial prefrontal cortex, paracentral lobule, thalamus, putamen and pons and decreases in the inferior frontal gyrus, angular gyrus, cingulate cortex and cerebellum in patients of PD with EDS (PD-EDS) (Wen et al., 2016; Wang et al., 2020; Zi et al., 2022; Zheng et al., 2023). Functional connectivity analysis can be used to evaluate correlations and synchronization at brain regions and the whole brain network levels. Multiple studies have demonstrated that EDS in PD patients may be associated with abnormal functional connectivity among various brain regions, including the default mode network (DMN), cognitive executive network (CEN), salience network (SN) and cerebellum (CB) (Wen et al., 2016; Ooi et al., 2019; Wang et al., 2020; Zi et al., 2022; Zheng et al., 2023). However, these static rs-fMRI data were measured under the assumption that the intrinsic fluctuations during MRI scans are stationary, ignoring the temporal dynamic properties of functional connectivity.

In fact, the functional connectivity of brain networks changes during rs-fMRI scanning sequences (Hutchison et al., 2013). Dynamic functional connectivity analysis can capture variability in connectivity among brain regions over short periods (Hutchison et al., 2013), providing greater insights from a time-varying perspective. In recent years, emerging evidence based on dynamic functional connectivity analyses has shown that abnormal dynamic properties are associated with motor and nonmotor symptoms in patients with PD. For example, the mean dwell time of the functional separation state was negatively correlated with the severity of motor symptoms (Kim et al., 2017) and positively correlated with the severity of cognitive impairment (Fiorenzato et al., 2019) in patients with PD. In addition, PD patients with the tremor-dominant subtype and PD patients with rapid eye movement sleep behavior disorder both had longer dwell times and sparser network connectivity than other PD patients without these symptoms (Gan et al., 2021; Zhu et al., 2021). Additionally, PD patients with depression preferred to remain in states characterized by DMN-dominated and CEN-disconnected patterns (Xu et al., 2021). A recent study demonstrated that PD patients with levodopa-induced dyskinesia were more frequently in and dwelled longer in strongly connected states, characterized by strong positive connectivity between the visual network (VIS) and sensorimotor network (SMN) (Si et al., 2023). Another study showed that PD patients with impulse control disorders preferred to be in states with brain configuration patterns characterized by sparse internetwork connectivity and strong intranetwork connectivity involving the limbic circuit (Navalpotro-Gomez et al., 2020). However, to date, the dynamic functional network connectivity (dFNC) characteristics and underlying neuropathological features of PD-EDS have remained unclear.

The purpose of this study was to identify dFNC characteristics in patients with PD-EDS to explain the potential neuropathological mechanisms underlying this condition. Considering the possible effect of drugs on the dynamic functional measures of patients with PD-EDS, we focused on drug-naïve PD patients in this study (Arnone et al., 2018; Si et al., 2023).

## Materials and methods

### Participants

The data used in this study were obtained from the Parkinson’s Progression Marker Initiative (PPMI) database, an ongoing, multicenter, international study aimed at identifying biomarkers related to PD progression. The inclusion and exclusion criteria have been published in PPMI (Marek et al., 2011).

**Figure 1:**
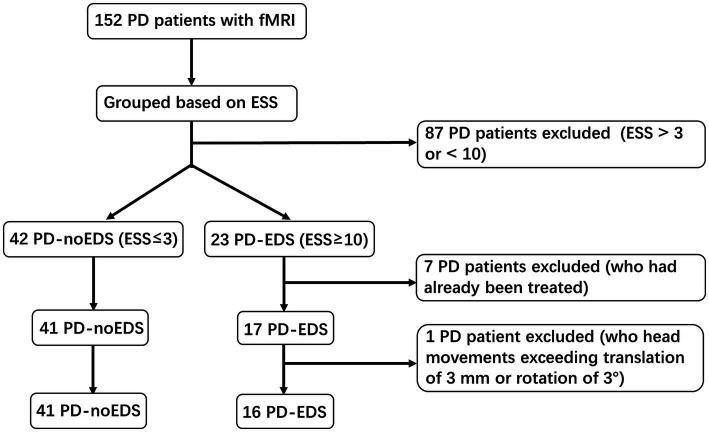
A flowchart of the selection of patients.

A total of 152 PD patients with functional magnetic resonance imaging (fMRI) scans acquired between December 2020 and March 2023 were recruited from the PPMI dataset. The inclusion criteria for our research were as follows: (1) patients had never taken anti-Parkinson’s medications or sleep-disrupting drugs before data collection; and (2) patients completed all clinical assessment scales and fMRI sequence scans. The exclusion criteria were as follows: (1) intracranial organic lesions, such as tumors, hematomas, and cerebral infarction; (2) patients with Parkinson’s syndrome or Parkinson’s superposition syndrome caused by other definite diseases; and (3) previous traumatic brain injury. The Epworth sleepiness scale (ESS) contains 8 items with scores ranging from 0 to 24 and is used to measure the subject’s expectation of drowsiness in 8 daily situations during the past 1 week. Based on the ESS scores, PD patients were divided into two groups. Patients with ESS scores of 10 or greater were classified as the PD-EDS group (*n* = 23), while those with ESS scores of 3 or less were classified as the PD patients without EDS (PD-noEDS) group (*n* = 42). Patients with ESS scores of 4 to 9 were excluded (*n* = 87) (Wang et al., 2020). In addition, seven patients with ESS scores ≤3 or ≥ 10 who had already been treated were excluded. Then, one PD-EDS patient was excluded due to its head movement exceeding translation of 3 mm or rotation of 3°in the later MRI data preprocessing. Finally, our study included 57 drug-naïve PD patients, including 16 PD-EDS patients and 41 PD-noEDS patients. A flowchart of the patient inclusion was provided in Figure 1.

### Clinical assessment

All patients were diagnosed by experienced neurologists. The patients’ age, sex, years of education, age at onset and duration of disease were recorded. All patients were evaluated with comprehensive clinical neuropsychological assessments including motor and nonmotor symptom-related scales, including the Unified Parkinson’s Disease Rating Scale III (UPDRS III), Hoehn and Yahr (H&Y) staging scale, Montreal Cognitive Assessment (MoCA), State–trait Anxiety Inventory (STAI), Geriatric Depression Scale (GDS), rapid eye movement sleep behavior disorder questionnaire (RBDSQ) and ESS.

### MRI data acquisition and preprocessing

All participants underwent rs-fMRI scans on 3.0 T MRI scanners (Siemens, Germany). Gradient-echo T2^∗^-weighted echo-planar imaging sequences (GE-EPI) were applied for functional brain activity imaging, and 240 time points were acquired during the scan. The rs-fMRI acquisition parameters were as follows: repetition time = 2,500 ms, echo time = 30 ms, flip angle = 80 degrees, thickness = 3.5 mm, voxel size = 3.5 mm^3^ and number of axial slices = 44.

The fMRI data were preprocessed using GRETNA software.^1^ To equilibrate the MRI signals and ensure subjects were adapted to the scanning environment, the first 10 time points were removed. The fMRI images acquired at the remaining 230 time points were then slice-time corrected and realigned to the mean echo-planar image. In addition, all corrected fMRI images were spatially normalized into the Montreal Neurological Institute (MNI) standard space using EPI templates, resampled with a 3 mm isotropic voxel, and spatially smoothed with a 6 mm 3D-Gaussian filter. To reduce the potential effects of head movements, we excluded patients with mean frame displacement (FD) > 0.5 mm or head movements exceeding a maximum translation of 3 mm or rotation of 3° (excluding one PD-EDS patient) from further analysis, referring to two previous studies (Navalpotro-Gomez et al., 2020; Xu et al., 2021). There were no significant group differences in head movements among the patients in the PD-EDS group (mean FD = 0.09 ± 0.06 mm) and the PD-noEDS group (mean FD = 0.11 ± 0.07 mm) (Mann–Whitney U test, *p* = 0.40).

### Group independent component analysis

Independent component analysis (ICA) was performed based on the preprocessed data using the group ICA in the functional MRI toolbox (GIFTv4.0b, http://mialab.mrn.org/software/gift/) to obtain specific data components. First, voxel-level variance normalization was performed based on all subject data. Next, the number of independent components (ICs) was estimated to be 36 on average using the minimum description length method. To improve accuracy, the subject data were reduced to 120 ICs using principal component analysis (PCA) and further decomposed into 50 components, as performed in previous studies (Si et al., 2023). Finally, the Infomax ICA algorithm in ICASSO was repeated 20 times to ensure stability and validity (Himberg et al., 2004). The spatial functional networks for each subject and corresponding time courses were created using the ICA back reconstruction method.

Among the 50 ICs, 27 ICs were identified as meaningful according to the following criteria by Allen et al. (2014), (1) peak coordinates of spatial maps located primarily in the gray matter; (2) low spatial overlap with known vascular, motion, and susceptibility artifacts; (3) time courses dominated by low-frequency signals (ratio of the integral of spectral power < 0.10 Hz to 0.15–0.25 Hz); and (4) time courses characterized by high dynamic range (range difference between the minimum and maximum power frequencies). These 27 ICs were then sorted into seven functional networks based on the spatial correlation values between the ICs and the template and the visual inspection results (Shirer et al., 2012). The seven functional networks were the auditory (AUD), basal ganglia (BG), cerebellum (CB), cognitive executive (CEN), default mode (DMN), sensorimotor (SMN), and visual (VIS) networks.

The time courses of the 27 ICs were postprocessed to remove remaining noise sources. The time courses were detrended with the 3DDESPIKE algorithm. Then, temporal bandpass filtering with a high frequency cutoff frequency of 0.15 Hz was performed (Allen et al., 2014). To obtain the static functional connectivity matrix, paired Pearson correlations were calculated and converted to z values before further analysis (Kim et al., 2017).

### Dynamic functional network connectivity

We used the k-means clustering algorithm in GIFT software and a sliding window to calculate the dFNC (Allen et al., 2014; Kim et al., 2017). To examine changes in the functional connectivity matrix across time, we created a sliding window of 45 s (width of 18 TRs) with a Gaussian distribution (σ = 3 TRs) in steps of 1 TR, resulting in 212 sliding windows per patient. We selected a window length of 45 s because the window size is approximately 30–60 s, this length could optimize the balance between the temporal resolution and the quality of the functional connectivity estimates (Li et al., 2014; Si et al., 2023). The windows for each dFNC matrix were obtained using paired Pearson’s correlation values between the time courses of the 27 ICs, forming a time series of FNC matrices (27 × 27) for the subsequent analysis. Next, all dFNC windows for each patient were classified by applying a k-means clustering method based on the Euclidean distance, which was repeated 100 times to reduce the bias of the random initialization of the centroid positions (Friedman et al., 2008). A k value of four (k = 4) was identified as the optimal cluster size using the elbow criterion. Furthermore, three different variables, including the fractional time, mean dwell time and number of transitions, were assessed to examine the temporal properties of the dynamic functional connectivity states. The fractional time was the percentage of time spent in one state. The mean dwell time was measured by averaging the number of consecutive windows in a specific state. The number of transitions was the number of conversions that occurred between different states.

The differences between the two groups (PD-EDS and PD-noEDS) were investigated using two-sample t tests or Mann–Whitney U tests. *p* values were corrected by the false discovery rate (FDR) for multiple comparisons. The threshold for statistical significance was set at *p* < 0.05.

### Statistical analyses

Statistical analyses were conducted using SPSS Statistic 24.0 (Chicago, IL, United States). Two-sample t tests or Mann–Whitney U tests were applied to compare the PD-EDS and PD-noEDS groups. Chi-squared tests were used to compare categorical variables such as sex. Spearman’s correlation analyses were conducted to assess the correlations between the detected temporal properties and the ESS scores (age and gender as covariates). Multiple comparison corrections were performed for dynamic functional connectivity parameters statistical analysis, and *p* < 0.05 with FDR correction was set as a threshold for statistical significance.

## Results

### Demographic and clinical characteristics

A total of 16 PD-EDS and 41 PD-noEDS patients were included in the analysis. There were no significant differences in demographics, disease duration and severity, cognition, anxiety, depression between groups. The ESS scores of the patients in the PD-EDS group were significantly higher than those of the patients in the PD-noEDS group (*p* < 0.001) (Table 1).

**Table 1:** Demographic and clinical characteristics of PD-EDS and PD-noEDS group.

	PD-EDS (*n* = 16)	PD-noEDS (*n* = 41)	*p*-value
Age	70.75 (58.93, 74.75)	62.00 (53.50, 70.60)	0.079^a^
Gender (M/F)	13/3	23/18	0.077^b^
Onset age (y)	68.45 (54.43, 72.45)	61.10 (51.70, 68.55)	0.077^a^
Disease duration (y)	2.45 (1.58, 3.83)	1.80 (1.10, 3.10)	0.324^a^
Education (y)	17.4 (14.5, 20.0)	18.0 (16.0, 20.0)	0.851^a^
H-Y staging	2 (1, 2)	2 (1, 2)	0.884^a^
UPDRS-III	24.50 (16.25, 32.50)	19.00 (14.00, 32.00)	0.374^a^
MoCA	26.5 (26.0, 29.0)	27.0 (25.5, 29.0)	0.815^a^
STAI-S	48.00 (44.25, 49.75)	47.00 (43.50, 50.00)	0.865^a^
STAI-T	45.94 ± 1.17	45.88 ± 0.56	0.957^c^
GDS	2.50 (1.00, 3.75)	1.00 (0, 2.50)	0.069^a^
RBDSQ	2.0 (1.0, 4.5)	3.0 (2.0, 4.5)	0.109^a^
ESS	12.00 (11.00,12.75)	2.00 (2.00, 3.00)	< 0.001^a^

Parametric variables are presented as mean ± SD, and non-parametric variables are presented as median (interquartile range). PD-EDS, Parkinson’s disease with excessive daytime sleepiness; PD-noEDS, Parkinson’s disease without excessive daytime sleepiness; M, Male; F, Female; y, Year; H-Y, Hoehn and Yahr scale; UDPRS-III, part III of the Unified Parkinson’s Disease Rating Scale; MoCA, Montreal Cognitive Assessment; STAI-S, state–trait anxiety inventory-state score; STAI-T, state–trait anxiety inventory-trait score; GDS, geriatric depression scale, RBDSQ, rapid eye movement sleep behavior disorder questionnaire; ESS, Epworth Sleepiness Scale. ^*^p < 0.05.

a Mann–Whitney U test.

b Chi-squared test.

c Two-sample *t*-test.

### Group independent components analysis

**Figure 2:**
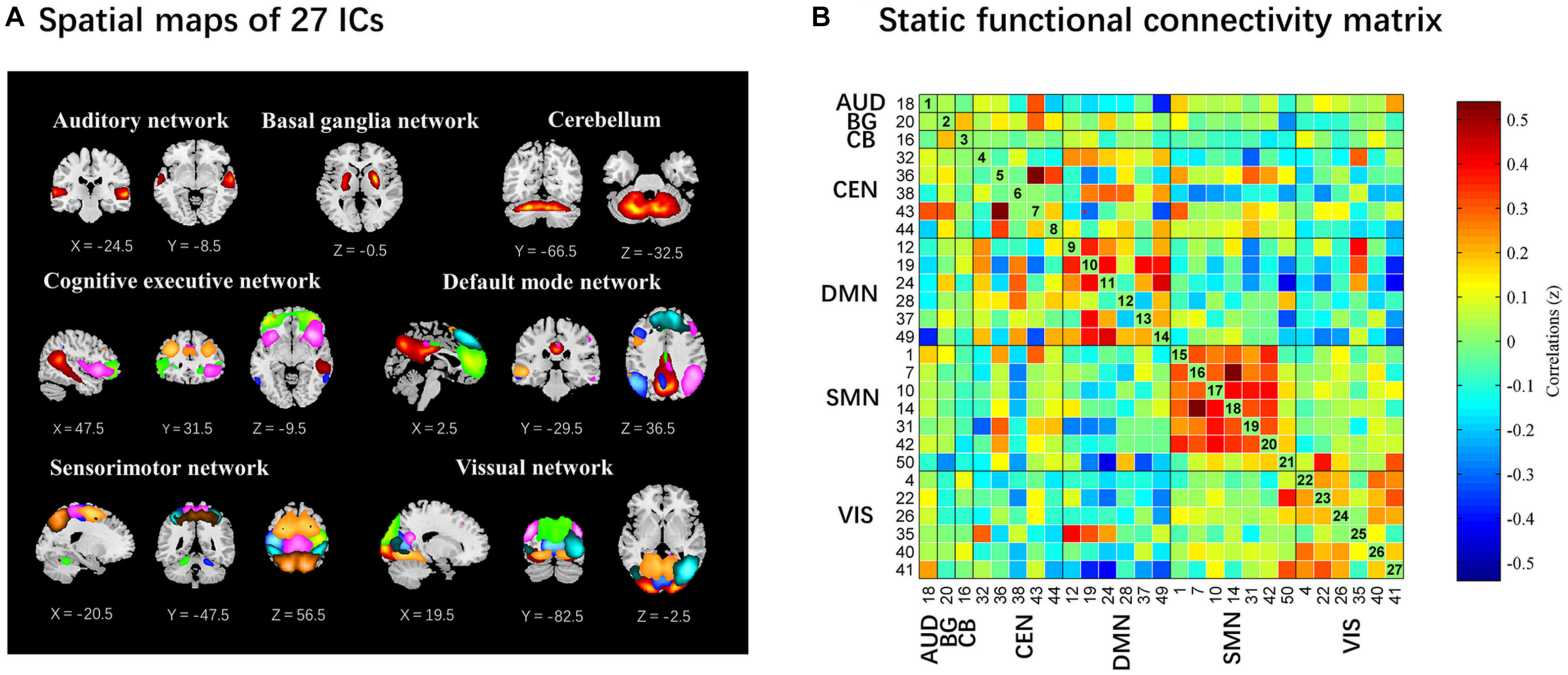
The 27 independent components identified by group independent component analysis. **(A)** Independent components spatial maps sorted into seven functional networks. **(B)** Group-averaged static functional connectivity between independent component pairs was computed using the entire resting-state data. The value in the correlation matrix represents the Fisher’s z-transformed Pearson correlation coefficient. Each of the 27 independent components was rearranged by network group based on the seven functional networks. AUD, auditory network; BG, basal ganglia; CB, cerebellum; CEN, cognitive executive network; DMN, default mode network; SMN, sensorimotor network; VIS, visual network.

The spatial map of all 27 ICs defined by using the group ICA is illustrated in Figure 2. These 27 ICs made up the following seven networks: auditory (AUD, IC 18), basal ganglia (BG, IC 20), cerebellum (CB, IC 16), cognitive executive (CEN, IC 32, 36, 38, 43, 44), default mode (DMN, IC 12, 19, 24, 28, 37, 49), sensorimotor (SMN, IC 1, 7, 10, 14, 31, 42, 50) and visual (VIS, IC 4, 22, 26, 35, 40, 41).

### Dynamic functional connectivity state analysis

**Figure 3:**
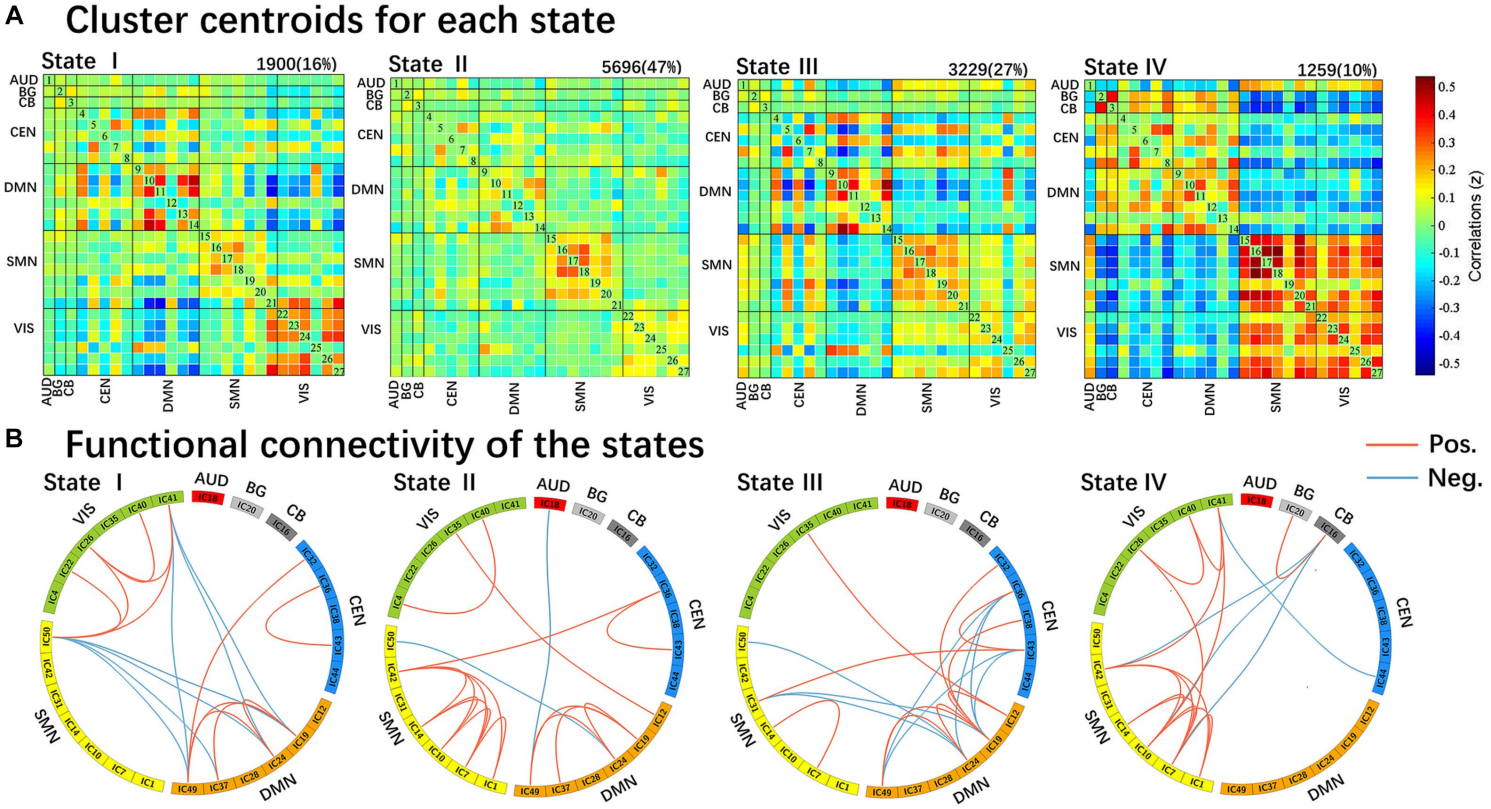
Results of the clustering analysis per state. **(A)** Resulting cluster centroids for each state. The total number of occurrences and percentage of total occurrences are listed above each cluster median. **(B)** Graphical representation of the strongest 5% functional network connectivity in each state. AUD, auditory network; BG, basal ganglia; CB, cerebellum; CEN, cognitive executive network; DMN, default mode network; SMN, sensorimotor network; VIS, visual network.

We identified four different dynamic functional connectivity states with the k-means clustering algorithm. Combined with the visualized clustering centroids, we retained the 5% strongest functional connectivity to better illustrate the connectivity characteristics of each state. As shown in Figure 3, state IV (10%) occurred least frequently and was characterized by strong positive correlations within and between the SMN and VIS, while strong negative connectivity were observed between the SMN and VIS with other networks (DMN/CEN/BG/CB). In addition, strong positive internetwork connectivity between the BG and CB were also observed in state IV. In contrast, the other three states (state I 16%; state II 47%; and state III 27%) occurred more frequently. State II occurred most frequently and was characterized by extensive sparse weak intra/internetwork connectivity. State I was characterized by strong positive connectivity intranetwork of the DMN and VIS, while negative connectivity internetwork between the DMN and VIS. For state III, strong positive connectivity within the DMN, positive and negative connectivity between DMN and CEN, and negative connectivity between DMN with SMN and VIS.

**Figure 4:**
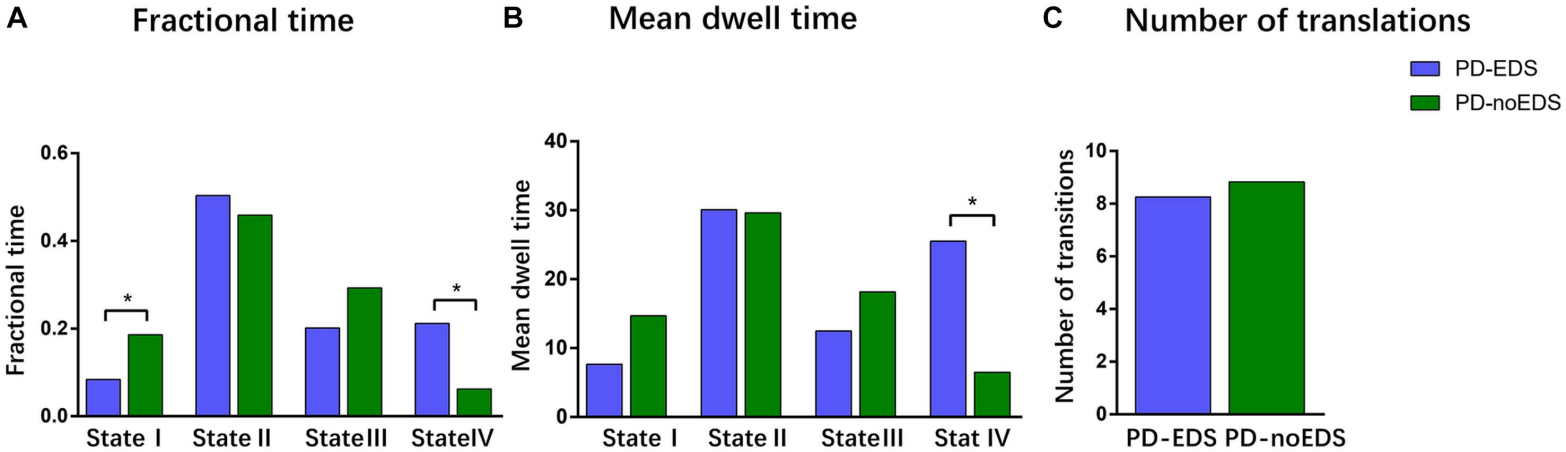
Differences in the temporal properties of the functional connectivity states between the two groups. **(A)** Fractional window spent by all subjects in each state as measured by percentage. **(B)** Mean dwell time between the two groups. **(C)** Number of transitions between the two groups. ^*^p < 0.05, PD, Parkinson’s disease; EDS, excessive daytime sleepiness; noEDS = without excessive daytime sleepiness.

We compared the group differences in the temporal properties of each state between the two groups (*p* < 0.05, FDR-corrected), as shown in Figure 4. We detected significant intergroup differences in the fractional time of state I and state IV and the mean dwell time of state IV. Specifically, the patients in the PD-EDS group had significantly higher fractional time and mean dwell time in state IV (*p* = 0.015, *p* = 0.027, FDR-corrected) and lower fractional time in state I (*p* = 0.022, FDR-corrected) than the patients in the PD-noEDS group. After controlling for the effects of age and gender, we found that the fractional time in state IV was slightly positively associated with ESS scores for all PD patients (*p* = 0.032, *r* = 0.289).

### Strength of the dynamic functional network connectivity states

**Figure 5:**
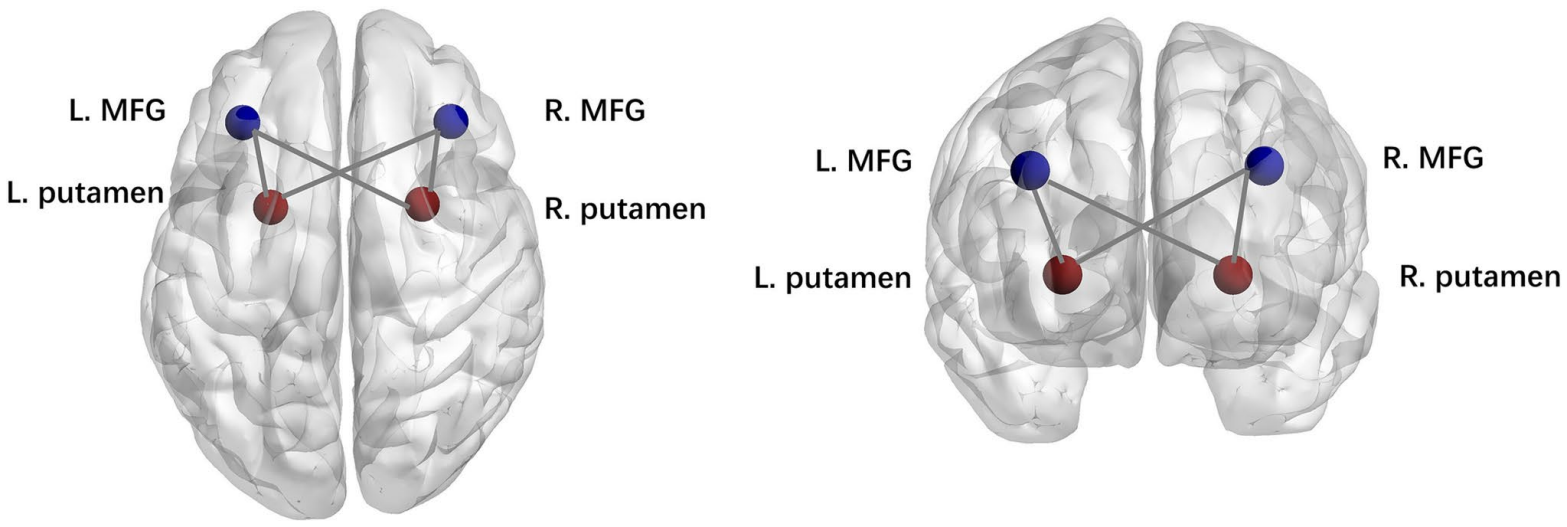
Strength of dynamic functional network connectivity (dFNC) in state II and state III. There were significant differences in state II and state III between PD-EDS and PD-noEDS groups (*p*<0.001, FDR-corrected) in strength of dFNC, located between the putamen and middle frontal gyrus.

We then examined the difference in the connection strengths in each state between the two groups. The results of the two-sample t test indicated that the patients in the PD-EDS group showed stronger dynamic connectivity between the bilateral putamen and middle frontal gyrus in state II and state III than the patients in the PD-noEDS group (*p* < 0.001, FDR-corrected), as shown in Figure 5.

## Discussion

In this study, dFNC analysis was used to explore the differences in dynamic functional network connectivity between the patients in the drug-naïve PD-EDS and PD-noEDS groups. Four recurrent distinct connectivity states were identified in both PD groups using a sliding time window approach. Interestingly, we observed that compared to the patients in the PD-noEDS group, the patients in the PD-EDS group showed significantly increased fractional time and mean dwell time in state IV and decreased fractional time in state I. The fractional time was positively correlated with EDS severity in state IV. Additionally, the patients in the PD-EDS group showed stronger dynamic connectivity between the bilateral putamen and bilateral middle frontal gyrus in state II and state III than the patients in the PD-noEDS group.

### Temporal properties of dynamic functional connectivity states

In our study, the patients in the PD-EDS group had significantly higher fractional time and mean dwell time in state IV (*p* = 0.015, *p* = 0.027, FDR-corrected) and lower fractional time in State I (*p* = 0.022, FDR-corrected) than the patients in the PD-noEDS group. This meant that PD-EDS patients frequently moved in and out of state IV and generally stayed in state IV for a longer period of time than PD-noEDS patients. Moreover, the fractional time was positively correlated with EDS severity in state IV. These findings showed that the state may be a characteristic state of EDS. State IV was characterized by dominant patterns in the SMN and VIS. Strong positive correlations within and between the SMN and VIS were mainly exhibited in state IV, while strong negative connectivity between SMN/VIS and other networks (DMN/CEN/BG/CB) were also be found in state IV. In addition, strong positive internetwork connectivity between the BG and CB were observed in state IV.

The SMN is involved in motor, auditory and sensory processing (Yang et al., 2021). Sensory information processing occurs during sleep. Different sensory modalities encoded by particular pathways or networks may modify physiological characteristics in the waking and sleep states (Velluti, 1997). In PD patients, some regions in the sensory network are associated with the formation of PD-EDS. Wen et al. found increased regional homogeneity values in the left paracentral lobule in early PD-EDS patients compared to PD-noEDS patients by using regional homogeneity analysis (Wen et al., 2016). In our study, we found that the patients in the PD-EDS group showed higher activity and information transmission in the SMN (high positive connectivity with the VIS and high negative connectivity with the CEN and DMN) over longer times than the patients in the PD-noEDS group, which was slightly positively correlated with EDS severity. These results suggest that the SMN increased the integration of visual processes and inhibited cognition and execution processes in patients with PD-EDS. Therefore, we concluded that patients with PD-EDS may exhibit enhanced SMN control to actively modulate sleep and wakefulness to compensate for the damage caused by EDS.

The VIS is one of the most important sensory networks. Previous neuroimaging studies have reported hypermetabolism in the visual cortex in narcolepsy patients (Dauvilliers et al., 2010; Huang et al., 2016; Dauvilliers et al., 2017) and increased functional connectivity in the visual network in adolescent narcolepsy patients (Fulong et al., 2020). These findings were similar to our results showing the strong positive connectivity within the VIS in state IV, and the fraction time in state IV was positively correlated with ESS scores. We inferred that one possibility is due to the patient’s subjective efforts to resist falling asleep during the scan, and the other may be a compensatory mechanism in the early stage of the disease. Notably, because this study included only drug-naïve PD patients with mild EDS, it is possible that this compensation occurs only in drug-naïve early PD-EDS. Our findings suggest that the fractional time in state IV is a potential valuable biomarker for PD-EDS.

In addition, in contrast to the other three states, strong positive internetwork connectivity between the BG and CB were observed in state IV. As a key function of the BG, the disruption of striatal neuron signaling may facilitate the occurrence of EDS symptoms in PD patients. In early PD patients, Ooi et al. showed that BG connectivity was positively correlated with ESS scores, which suggested the existence of compensatory mechanisms in early PD patients (Ooi et al., 2019). The CB may also be associated with EDS symptoms in PD patients. Compared with the patients in the PD-noEDS group, the patients in the PD-EDS group had decreased connectivity within the CB, decreased connectivity between the CB and the insula, frontal lobe, and BG regions, and enhanced connectivity with the postcentral gyrus (Wen et al., 2016; Ashraf-Ganjouei et al., 2019; Zi et al., 2022; Zheng et al., 2023). Strong functional connectivity was observed between the CB and BG after sleep deprivation, which was thought to be a natural response to the decrease in motor alertness (Zhang et al., 2019). In our study, the dFNC analysis results demonstrated that interactions between the BG and CB were associated with the emergence of EDS in PD patients and that this strong positive connectivity may be attributed to early compensatory mechanisms.

In our study, compared to the patients in the PD-noEDS group, the patients in the PD-EDS group showed significantly decreased fractional time in state I. This meat that PD-EDS patients were more stable when they stayed in state I. State I was characterized by strong positive connectivity intranetwork of the DMN and VIS, while negative connectivity internetwork between the DMN and VIS. The DMN is a highly integrated task-negative network that is activated when people are in waking resting states and is responsible for conscious awareness and self-referential introspective states (Qin et al., 2015; Mak et al., 2017). A study focusing on early PD-EDS reported a positive correlation between the occurrence of EDS and DMN connectivity (Ooi et al., 2019), the same with our findings, suggesting the presence of compensatory mechanisms in the early stages of the disease. As mentioned above, the VIS is one of the most important sensory networks. The VIS also showed an intranetwork strong positive connectivity in state I. These findings suggested that PD-EDS patients may achieve a more stable state (state I) by strengthening the internal regulation of DMN and VIS. However, we found state I was also characterized by negative connectivity internetwork between the DMN and VIS. This may be an interesting finding. Judging from the strong negative connectivity between the DMN and VIS in state I, the DMN and VIS were functionally competitive and inhibitory networks. Therefore, we speculated that this may be due to the competition between the DMN and VIS networks for brain resource allocation when maintaining brain stability. Nevertheless, this finding needs to be further verified by future studies.

### Strength of dynamic functional connectivity states

We observed that the patients in the PD-EDS group showed stronger dynamic connectivity between the putamen and middle frontal gyrus in state II and state III than the patients in the PD-noEDS group (*p* < 0.001, FDR-corrected). As mentioned above, compensatory mechanisms in the striatum may be related to EDS symptoms in PD patients (Gong et al., 2019). The putamen is one of the structures of the striatum and is an important node in various neural circuits and neural networks. Additionally, fALFF values in the putamen and functional connectivity between the putamen and medial frontal gyrus were elevated in PD-EDS patients (Zheng et al., 2023). These findings suggested that EDS symptoms in PD patients are associated with abnormal information integration between the putamen and middle frontal gyrus. Our study supports this view from the perspective of dynamic functional connectivity analysis.

### Limitations

This study has some limitations that should be considered. First, as in previous studies, head motion has an unavoidable effect on rs-fMRI data. Although we carried out several manipulations to mitigate this effect, it was still not completely ruled out. Second, in this study, the sample size of patients in the PD-EDS group was relatively small and there were differences in gender between the two groups (PD with and without EDS group). Future studies with larger sample sizes should be performed for further validation. In addition, we did not collect detailed information on patients’ sleep habits or quality, which prevented us from assessing whether nocturnal sleep fragmentation or other sleep difficulties associated with PD impacted our analysis (Tandberg et al., 1998; Videnovic and Golombek, 2013). However, there was no significant difference in RBDSQ scores between the two groups, which ruled out the effect of rapid eye movement sleep behavior disorder in PD patients to some extent.

## Conclusion

In summary, this study is the first to examine the dynamic functional network connectivity characteristics of PD-EDS. The dynamic functional connectivity analysis showed that PD-EDS patients preferred to be in states dominated by the SMN and VIS, with strong positive correlations within and between the SMN and VIS, while strong negative connectivity between the SMN/VIS with other networks. These findings revealed that SMN-and VIS-dominated patterns were specific network aggregation states associated with PD-EDS. Moreover, the duration of this state correlated with the severity of EDS, suggesting that SMN-and VIS-dominated patterns may compensate for EDS damage through high-intensity information output. Our research provided new insights into the neural mechanisms underlying PD-EDS, and the SMN-and VIS-dominated patterns may serve as biomarkers of the pathophysiological features of PD-EDS.

## Data availability statement

The datasets presented in this study can be found in online repositories. The names of the repository/repositories and accession number (s) can be found at: https://www.ppmi-info.org/.

## Ethics statement

The studies involving humans were approved by the respective ethics committees of the different data collection centers on PPMI. The studies were conducted in accordance with the local legislation and institutional requirements. Written informed consent for participation was not required from the participants or the participants' legal guardians/next of kin in accordance with the national legislation and institutional requirements. Written informed consent was obtained from the individual(s) for the publication of any potentially identifiable images or data included in this article.

## Author contributions

ZT: Data curation, Formal analysis, Investigation, Methodology, Software, Writing – original draft. QZ: Data curation, Formal analysis, Investigation, Methodology, Software, Supervision, Writing – review & editing. XH: Data curation, Writing – review & editing, Visualization. DD: Data curation, Writing – review & editing. LC: Data curation, Writing – review & editing. ZL: Conceptualization, Funding acquisition, Investigation, Resources, Writing – review & editing. GC: Conceptualization, Funding acquisition, Resources, Project administration, Supervision, Writing – review & editing.

## Footnotes

^1^https://www.nitrc.org/projects/gretna/
